# Role of human papillomavirus and its detection in potentially malignant and malignant head and neck lesions: updated review

**DOI:** 10.1186/1758-3284-1-22

**Published:** 2009-06-25

**Authors:** Ajay Kumar Chaudhary, Mamta Singh, Shanthy Sundaram, Ravi Mehrotra

**Affiliations:** 1 Centre for Biotechnology, University of Allahabad, India; 2 Department of Pathology, MLN Medical College, Allahabad, India

## Abstract

Head and neck malignancies are characterized by a multiphasic and multifactorial etiopathogenesis. Tobacco and alcohol consumption are the most common risk factors for head and neck malignancy. Other factors, including DNA viruses, especially human papilloma virus (HPV), may also play a role in the initiation or development of these lesions. The pathways of HPV transmission in the head and neck mucosal lesions include oral-genital contact, more than one sexual partner and perinatal transmission of HPV to the neonatal child. The increase in prevalence of HPV infection in these lesions may be due to wider acceptance of oral sex among teenagers and adults as this is perceived to be a form of safe sex. The prevalence of HPV in benign lesions as well as malignancies has been assessed by many techniques. Among these, the polymerase chain reaction is the most sensitive method. Review of literature reveals that HPV may be a risk factor for malignancies, but not in all cases. For confirmation of the role of HPV in head and neck squamous cell carcinoma, large population studies are necessary in an assortment of clinical settings. Prophylactic vaccination against high-risk HPV types eventually may prevent a significant number of cervical carcinomas. Of the two vaccines currently available, Gardasil^® ^(Merck & Co., Inc.) protects against HPV types 6, 11, 16 and 18, while the other vaccine, Cervarix^® ^(GlaxoSmithKline, Rixensart, Belgium) protects against HPV types 16 and 18 only. However, the HPV vaccine has, to the best of our knowledge, not been tried in head and neck carcinoma. The role of HPV in etiopathogenesis, prevalence in benign and malignant lesions of this area and vaccination strategies are briefly reviewed here.

## Background

Head and neck squamous cell carcinoma (HNSCC) is a significant cause of cancer worldwide [1]. Incidence rates of these malignancies have been rising in most regions of the world. It is estimated that 35,310 (25,310 males and 10,000 females) new cases of oral cavity & pharyngeal malignancies will be diagnosed in the US during 2008, while 7,590 (5,210 males and 2,380 females) patients will die of the disease [2]. The authors reported that oral and oropharyngeal malignancies were commonest in men in North India and accounts for about 30–40% of all cancer types in India making it a leading cause of cancer mortality [3-5]. The term "head and neck cancer" has been widely adopted in the recent literature and includes lesions at several anatomical sites: lip, oral cavity, nose and paranasal sinuses, nasopharynx, oropharynx, hypopharynx, and larynx [6]. They are characterized by a multiphasic and multifactorial etiopathogenesis [7-9].

Tobacco and alcohol consumption are the most common risk factors for HNSCC [10]. In addition, Bosh et al reported that approximately 15% of all malignancies worldwide appear to be connected with viral infections and several human DNA viruses are now accepted as causative factors [11]. Majority of the head and neck malignancies originate from the epithelium which lines the upper aerodigestive tract i.e., the oral cavity, pharynx and larynx [12,13]. The epithelial areas of the upper aero-digestive tract display greatest susceptibility to HPV due to the easy exposure of the basal cells to HPV infection [14]. The pathways of HPV transmission in the mucosal lesion may be oral-genital contact by kissing and oral sex, more than one sexual partner and perinatal transmission of HPV to the neonatal child [15,16].

The participation of HPV in oral and oropharyngeal carcinogenesis was first proposed by Syrjanen et al in 1983 [17] and then supported by several other authors [18-21] including Gillison et al who reported that the high-risk human papillomavirus (HR-HPV) genotypes are involved in a subset of HNSCC in their epidemiological and molecular study [22].

Many detection methods have been used to study the role of HPV virus, like in-situ hybridization (ISH), Immunohistochemistry (IHC), Southern Blotting, Polymerase Chain Reaction (PCR), DNA micro-arrays and Hybrid Capture-II (HC-II) high risk and low risk detection methods. In the PubMed database, the following keywords were used for the literature survey: HPV detection methods, HPV in head and neck cancer, HPV in benign lesion, premalignant and malignant lesion of head and neck, HPV in oral pre-cancer, HPV in oral cancer, HPV in larynx, HPV in Pharynx, Expression of HPV in OSMF, detection of high risk HPV by PCR etc. The objective of this review is to summarize the various techniques of detection and the role of HPV in the etiology of benign, potentially malignant and malignant lesions of head and neck.

### Molecular structure and role of high and low risk HPV in HNSCC

Human Papilloma virus is about 55 nm in diameter. It has a single circular double stranded DNA molecule and belongs to the family papillomaviridae. Its genome is made up of 7,200 – 8,000 base pairs with a molecular weight of 5.2 × 10^6 ^D. On the basis of DNA base pair (bp) distribution, the viral DNA is divided into three parts: first a 4,000 bp region that responsible for viral DNA replication and cell transformation, second 3,000 bp region that encodes the structural proteins of the virus particles and last 1,000 bp non-coding region (NCR) that contains the origin of viral DNA replication.

**Figure 1:**
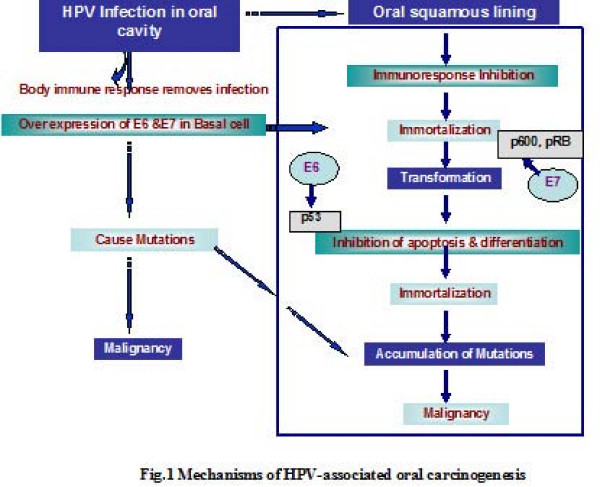
**Mechanisms of HPV-associated oral carcinogenesis**.

Zur et al suggested that the genomic HPV DNA has nine open-reading frame sequences (ORFS) -present on single strand of DNA and are divided into seven early (E) and two late-phase genes (L). The transcription of viral DNA is regulated by early phase gene, while the capsid proteins (involved in viral spread) are regulated by late phase gene [23]. The early-phase gene (E) encodes the E1, E2, E5, E6, and E7 proteins. E1 and E2 gene products regulated the transcription and replication of viral proteins and E5 gene product transcribed from the episomal region of the viral DNA [24]. The E6 and E7 oncoprotiens are usually under control of E1 and E2 inhibitory genes. These genes have the ability to de-stimulate the tumor suppressor function and regulate the functions of the p21, p53, and pRb proteins, resulting in apoptosis, DNA repair and cell cycle control and may finally lead to cellular immortalization. (Fig. 1) The non-coding, long control region (LCR) contains binding sites for the E1 and E2 gene products, located just upstream of the promoter sequence 97 (P 97) which controls the transcription of the E6 and E7 oncogenes [25].

HPV must adhere to a specific receptor protein on the keratinocytes membrane. Once the virus entered into the cell, it transforms itself of its protein coat and the viral DNA may then utilize host cell themselves. These viruses elaborate early gene proteins (E) that are able to regulate the host cell cycle, or mitotic capabilities. The E6 and E7 proteins are most important in this respect; they bind two host proteins that are regulators of the keratinocytes at the time of cell division. E6 binds to a protein designated p53, a molecule that arrests cell division. However, once bound, it is degraded and this inhibition of keratinocytes mitosis is abrogated. Likewise, E7 binds a protein termed Rb; and, similarly, cell cycle regulation is troubled.

On the basis of their genotype, more than 120 types of HPV viral DNA genotypes have been fully sequenced [26,27]. It classified on the bases of their infection in epithelial cells and the ability to effect cellular transformation for e.g. HPV 1 is responsible for the infection in cutaneous cell while HPV 6, 11, 18 for mucosal epithelial cells of the oral cavity, oropharynx, anogenital tract and uterine cervix [28]. The potentially oncogenic HPV is divided into high and low-risk types. The high-risk HPV such as 16, 18, 31, 33, 35, 52, 58, 59, 68, 73, and 82 are responsible for malignancies while the low-risk sub types (6, 11, 40, 42, 43, 44, 54, 61, 70, 72 and 81) are rarely found in carcinoma and frequently connected with benign and potentially malignant lesions of the head and neck.

Although HNSCC is closely linked to tobacco and alcohol use, there is an increasing incidence HPV16-related HNSCC arising in the oropharynx [29,22,31]. Bouda et al suggested that high risk HPV E6/E7 transcripts and viral integration have also been detected in HNSCC. Transcriptionally active HR-HPVs, particularly HPV-16, are found in a subset of HNSCC. HPV16-associated carcinogenesis is mediated by expression of the viral E6 and E7 oncoproteins, which cause deregulation of the cell cycle by inactivating p53 and pRb respectively [26]. HNSCC with transcriptionally active HPV16 DNA are characterized by occasional chromosomal loss, whereas HNSCCs lacking HPV DNA are characterized by gross deletions that involve whole or large parts of chromosomal arms and that already occur early in HNSCC development. These distinct patterns of genetic alterations suggest that HPV16 infection is an early event in HNSCC development [32]. Chen et al suggested that the combined variants of p53 and p73 significantly increase the risk of HPV16-associated oral cancer, especially among never-smokers [33]. In another report, they studied the p73 G4C14-to-A4T14 polymorphism may modulate the risk of HPV-16-associated oropharynx squamous cell carcinoma (SCCOP) and the p73 variant genotypes may be markers of genetic susceptibility to HPV-16-associated SCCOP, particularly in never smokers and never drinkers [34]. Ji et al reported that p73 polymorphism was connected with HPV-16 in HNSCC and may serve as a marker of positive HPV-16 tumor in patients with HNSCC, in oropharyngeal cancer [35]. Li et al suggested that this p73 polymorphism may be a risk marker for genetic susceptibility to HNSCC [36].

Pintos et al reported that HPV 16 was identified in tonsil-related cancers. [37]. HPV has been found to be the most significant positive prognostic factor in patients with oropharyngeal tumors, with a 60–80% reduction in the risk of death [38]. HPV positivity designates a specific subgroup of oropharyngeal squamous cell carcinomas that arise preferentially among individuals with no consumption of tobacco and alcohol and that have a favorable outcome attributable to an increased sensitivity toward radiotherapy [39].

### Detection Techniques

#### Pathological Examination

#### Cytological and Histopathological Examination

**Figure 2:**
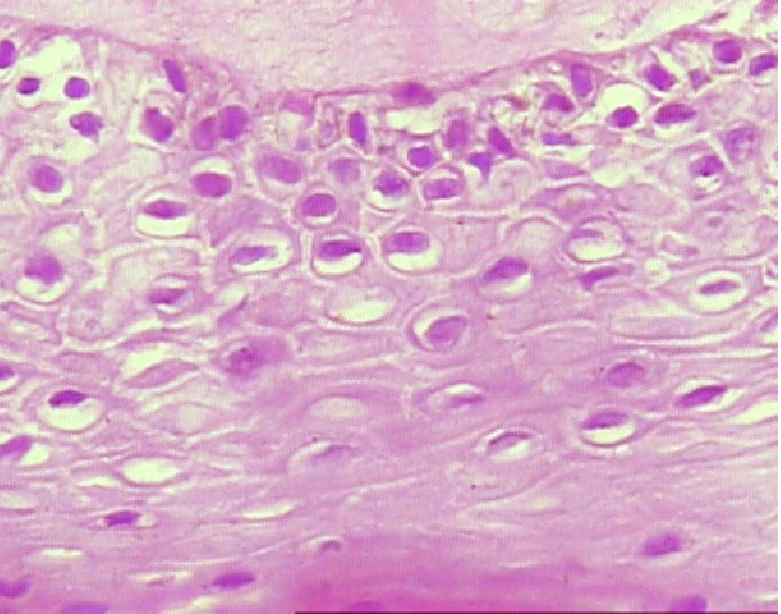
**Squamous Epithelial layer showing koilocytosis**.

Different methods are used to detect specific types of HPV DNA in lesions and shows varying sensitivity and specificity [40]. The detection of HPV in the oral mucosa may be done by cytology and histological examination. On the basis of cytology and histopathology, HPV infection is characterized by koilocytosis, perinuclear cytoplasmic haloes, nuclear dysplasia, atypical immature metaplasia and binucleation. (Fig. 2) These methods show limited sensitivity and are unable to determine which types of HPV are involved in the infection of the epithelial cells. Smith et al suggested that oral scrapes or rinse samples, with their greater surface area of mucosa than with biopsies, are less invasive, and high risk HPV detection in oral exfoliated cells is a reliable biomarker of an HPV-related head and neck cancer risk. A drawback is that not all patients who have HR-HPV types in oral exfoliated cells are detected with HPV DNA in the primary tumour [31].

#### In situ hybridization (ISH) and immunohistochemistry

In situ hybridization (ISH) techniques employ the use of type-specific radioactively labeled DNA probes, which are complementary to HPV DNA sequences used for detection of viruses in the premalignant and malignant lesions of the head and neck. ISH and immunohistochemistry have low sensitivity because these tests only detect the virus when it is present in more than 10 copies of the viral DNA per cell. Lee et al studied the formalin-fixed, paraffin-embedded tissue sections through in situ hybridization using newly available probe sets and concluded that the new probe sets for HPV can be used very efficiently in clinical pathology material of HNSCC [41].

### Molecular detection methods

#### Southern Blotting

Southern Blotting is an assay that has long been one of the standard techniques for the detection of HPV DNA. This blotting has the ability to differentiate between episomal and integrated DNA and can detect up to 0.1 copy of viral DNA per cell. This method has some technical variability and requires a significant amount of DNA. Frazer and Kay et al suggested that Southern blot may boast a theoretically higher specificity but is clearly less sensitive than PCR [42,43]. Similarly; Gillison et al studied the Southern blot and reported that, in non-oropharyngeal tumors, it was rarely positive when compared with PCR. [22]. Yeudall et al utilized both type-specific PCR and Southern blot for HPV 16/18 and reported that there was a manifest variation in the two techniques. Two of 39 oral carcinoma samples were positive for either HPV 16/18 by Southern blot, whereas 18/39 was positive by type specific PCR for HPV 16/18. These studies evidently demonstrate the difference in sensitivity in these two assays. [44].

#### Polymerase chain reaction (PCR)

PCR is highly sensitive detection method for specific subtypes of HPV because it detects the virus in less than 1 copy of the viral DNA per cell [45]. Universal primers of conserved DNA sequences in HPV have been designed to the L1 region i.e. MY09/MY11 [46], E1 region i.e. CPI and CPII, E6 region and E7 region [47]. The majority of studies used the primers MY09/11 for the detection of human papilloma virus having base pair 450 bp responsible for different types of cancers.

Quantitative PCR utilizes a fluorescent probe which helps to measure the degree of fluorescence in the reaction mixture. Ha et al reported the advantages of quantitative PCR in premalignant and malignant lesions of the oral cavity and analyzed the samples previously found to be HPV positive by other molecular methods [21]. Termine et al conducted a meta-analysis of studies (1988–2007) on HPV in non site-specific HNSCC vs. OSCC biopsies and found that the pooled prevalence of HPV DNA in the overall samples was 34.5%, while it was 38.1% in OSCC and 24.1% in the non site-specific HNSCC group [48]. Kreimer et al also studied the genotype frequency of HR-HNSCC and reported that an overall prevalence of HPV in 25% in HNSCC, 35.6% in oropharyngeal cancer and 23.5% in OSCC [49].

#### Hybrid capture II (HC II)

The HC II technique is a nucleic acid hybridization assay with signal amplification that utilizes microplate chemiluminescent detection. First, double stranded DNA is denatured by using a strong alkaline denaturation solution that converts it into single stranded DNA (ssDNA). This ssDNA is then hybridized in-solution to a cocktail of specific 13 high risks HPV RNA probes. The resultant DNA-RNA hybrids are captured onto the surface of a micro-well plate coated with particular antibodies for DNA-RNA hybrids. The immobilized hybrids are reacted with alkaline phosphate conjugated antibody and detected by cleavage of the chemiluminescent substrate. The emitted light is measured as relative light unit (RLU) in a luminometer. The intensity of the light is proportional to the amount of target DNA in the sample. Specimens with RLU greater than or equal to the mean of the three positive control values are considered HPV positive.

Estimation of HPV Viral Load, a ratio of relative light unit (RLU) of the sample to mean of RLU of the three positive controls (PC), has been taken as estimate of approximate viral load, which relates to the index of intensity of HPV infection. This ratio of any specimen represents empirically a relative measure of its viral load. Cut off value (RLU of specimen/mean RLU of PC) is 1.0 pg/ml of sample. The hybridization method of HC II Digene^® ^is approved by Food and Drug Administration (FDA) USA for routine clinical purposes. Detection of HPV DNA is carried out by using probes of 13 high risks HPV genotypes (16, 18, 31, 33, 35, 39, 45, 51, 52, 56, 58, 59 and 68).

#### Gene Expression: DNA Microarray

DNA microarray is a compilation of microscopic DNA spots on a solid surface by covalent attachment to a chemical matrix. Each gene on the solid supports referred to as spot or probe is usually less than 200 μm in diameter. Each spot has a unique sequence different from the others in the array and will hybridize only to its complimentary strand. This technique uses a DNA probe labeled with either a radioisotope or a fluorescent tag. The probe is applied to the fragment of DNA or RNA to be studied and by the rules of base pairing (A to T, C to G) "sticks" to its complementary sequence. This technology has made it possible to miniaturize methods of probe detection for DNA and allow detection of several thousand DNA or RNA sequences in one experiment.

DNA microarrays have been successfully used to identify global patterns of gene expression in different human neoplasia, including head and neck cancers. [50]. Many investigators have used microarrays to analyze gene expression changes in HNSCC in tissues and cell lines, but little is known about the gene expression changes in HPV associated HNSCC [51-53]. The identification of molecular portrait of gene expression profiles in HPV-positive and negative HNSCC, including their differences, could result in a better understanding of critical events during carcinogenesis.

Ivan et al compared the cellular gene expression profiles of HPV-positive and negative in normal epithelium and oropharyngeal carcinomas with the help of Affymetrix Human U133A Gene Chip^® ^and suggested the specific expression of gene patterns in HPV-positive and HPV-negative oropharyngeal squamous carcinomas that may serve as potential biomarkers for the development of HNSCC [54]. DNA microarray technology is an exciting, rapidly expanding biotechnological methodology and will hopefully enable both a greater understanding of the biology of head and neck cancer and help in the clinical management of this disease.

### Prevalence of HPV in benign lesions of the Head and neck

Detection methods of HPV in benign lesions indicates that not all types of viral infections can cause disease and it would be essential to recognize the factors that determine its capability to induce malignant transformation in head and neck lesions. Respiratory papillomatosis associated with human papilloma virus (HPV) infection is the most common benign laryngeal neoplasm [55]. Laryngeal papillomatosis is the most frequent benign neoplasia in children and it is caused by HPV 6 and 11. The lesions are exophytic and highly recurrent, compromising the airway mucosa, mainly the larynx [56]. Juvenile laryngeal papillomatosis is a rare condition caused by HPV [57]. DeVoti et al reported that recurrent respiratory papillomas (RRP) are benign airway tumors, caused primarily by HPV types 6 and 11 [58]. Castro et al reported the prevalence of HPV 6, 11 in normal oral mucous membrane. In oral benign lesions associated with HPV, a prevalence of HPV 6 and 11 was observed in squamous cell papilloma (SCP) and condyloma acuminatum, while HPV 2 and 57 were more prevalent in verruca vulgaris lesions [59]. Fischer et al suggested that the applied broad-spectrum PCR system is a reliable tool in the detection of HPV DNA in benign lesions of the upper aerodigestive tract [60]. Hoffmann et al suggested that HPV is correlated to a lesser extent to infectious mucosal lesions than to proliferative lesions. Furthermore, the results emphasize that the presence of HPV in specific lesions does not occur by chance, but represents a specific infection of the mucosa leading to proliferation and even to malignancy [61]. (Table 1)

**Table 1:** Prevalence of HPV identification in benign lesions of the Head and neck

**Study**	**Year**	**HPV type**	**Mode of Detection**	**Tumor Type**
Fischer et al [60]	2005	Different HPV types	Applied broad-spectrum PCR	Benign lesions

Castro et al [59]	2006	6/11	Review	Squamous cell papilloma

Martins et al [56]	2008	6	HPV typing (PCR), light microscopy and electron microscopy	Laryngeal papillomatosis

Förster et al [57]	2008	6/11	PCR	Juvenile laryngeal papillomatosis

DeVoti et al [58]	2008	6/11	PCR, micro array	Respiratory papillomas (RP)

Kovalenko et al[55]	2009	6/11	IHC	Respiratory papillomatosis

### Prevalence of HPV in potentially malignant lesions of the Head and neck

Several investigators have studied the prevalence of HPV in potentially malignant lesions, hoping to find a similar HPV prevalence as in malignant diseases. An increasing prevalence of HPV in premalignant lesions suggests that it may play a role in malignant transformation. Ha et al reported 1% prevalence of HPV 16 and 18 in premalignant oral lesions by using the quantitative PCR. [21]. On the other hand, Bagan et al did not find any association between HPV DNA an PCR in proliferative verrucous leukoplakia (PVL) and HPV [62].

Furrer et al reported 41% HPV DNA detection by PCR-Southern blot analysis in premalignant and malignant lesions [63]. Ostwald et al studied the occurrence of DNA of different subtypes of HPV 6/11, 16 and 18 detected by using highly sensitive molecular techniques like PCR/Southern blot hybridization and reported that 22.2% oral leukoplakias (OL) and 15.4% oral lichen planus (OLP) lesions were positive in HPV DNA [64]. Campisi et al studied the prevalence of HPV infection in OL and OLP in comparison with healthy oral mucosa and reported that HPV DNA was found in 17.6% of OL and in 19.7% of OLP [65]. Luo et al reported that prevalence of HPV was 30.4% in precancerous lesions through nested PCR and gene-chip arrays [66]. (Table 2)

**Table 2:** Prevalence of HPV identification in premalignant lesions of Head and Neck

**Study**	**Year**	**Positive/cases**	**%**	**Lesion Type**	**Mode of Detection**
Zeuss et al [87]	1991	0/20	0	Dysplasia	ISH 6/11, 16/18, 31/33/35

Holladay & Gerald [88]	1993	13/45	28.9	Dysplasia, inflammation hyperplasia	E1 PCR

Fouret et al [89]	1995	0/3	0	Dysplasia	E6 consensus PCR

Nielsen et al [90]	1996	20/49	40.8	Dysplasia leukoplakias	ISH/HPV 16 PCR, SB PCR

Wen et al [91]	1997	5/17	29.4	Papilloma, leukoplakias, OLP	E6 HPV 16/18 PCR

D'Costa et al [70]	1998	27/80	33.8	Leukoplakias, OLP, OSMF	L1 consensus PCR

Bouda et al [26]	2000	29/34	85.2	Hyperplasia dysplasia	Nested consensus PCR

Ha et al [21]	2002	1/102	1.0	Dysplasia	Quantitative PCR

Ostwald et al [64]	2003	72/118	22.2	Oral Leukoplakia	PCR/Southern blot hybridization
		65/118	15.4	OLP	

Lo Muzio et al [92]	2004	6/7	85.7	Oral leukoplakia	Nested PCR, IHC

Campisi et al [65]	2004	68/139	17.6	Oral Leukoplakia	PCR
		71/139	19.7	OLP	

Chen et al [67]	2006	12/23	52.6	OSMF	PCR

Furrer et al [63]	2006	9/22	41	Potentially malignant and malignant head and neck lesions	PCR- Southern Blot

Giovannelli et al [93]	2006	50/116	22	Oral leukoplakia	PCR

Bagan et al [62]	2007	0/13	0	Proliferative verrucous leukoplakia	PCR

Luo et al [66]	2007	14/46	30.4	Pre-cancerous lesion	Nested PCR, gene-chip arrays

Recently, a study was conducted by the author's group with the aim to analyze the high-risk HPV DNA in oral submucous fibrosis (OSMF) and to determine the role of HPV infections. 105 cases, 33(31.4%) patients were positive for high risk HPV, while 72 (68.6%) were negative. On correlating HPV with histopathological grading, 7(29.1%) patients of grade I, 11(28.9%) of grade II and 15(34.8%) of grade III OSMF were positive. While correlating addiction habits with HPV, 18 (34.6%) areca nut chewers were positive and showed a significant p value (<0.05). The results showed that the level of agreement between the HPV and koilocytosis was moderate, with a Cohen kappa value of 0.524 (95% confidence interval [CI]. 0.3406 to 0.7081). The sensitivity of the hybrid capture test vis-à-vis presence of koilocytosis was 59% (95% CI: 0.4427 – 0.7363) and specificity was 91% (95% CI: 0.8102 – 0.9714) while the positive predictive value was 84% (95% CI: 0.6810 – 0.9489) and negative predictive value was 73% (95% CI: 0.6190 – 0.8330 (Unpublished data). Chen et al reported the positivity of HPV16 as 52.6% and HPV 18 as 25% in OSMF by the PCR method [67]. Vidal et al reported 72.5% negative for low and high risk HPV DNA, 22.5% positive for low and high risk HPV DNA, 2.5% positive for low risk HPV DNA and 2.5% positive for high risk HPV DNA in 40 oral carcinoma cases by the Hybrid Capture technique [68].

### Prevalence of HPV in malignant lesions of the Head and neck

Human papillomavirus especially the high-risk (HR) types (16 and 18), are widely recognized in the promotion of tumorigenesis in the uterine cervical squamous cell carcinoma [18]. High-risk HPV are predominantly found in OSCC and may play a role in its progression, while low-risk sub types are usually associated with oral precancerous lesions. Koppikar et al studied the HPV DNA in oral carcinoma by PCR amplification and reported that HPV could contribute to carcinogenesis [69].

D'Costa et al suggested that HPV 16 plays a direct role in a certain populations of oral cancers whereas in a subpopulation, HPV16 infection may be vital in the early events associated with development of potentially oral lesion to malignancy [70]. Mishra et al reported that involvement of p65 in HPV infected oral cancer may be linked to improved differentiation and better prognosis of the disease [71]. König et al suggested that over expression of p16 has been demonstrated to be strongly related to the presence of HPV16, 18. HNSCC has been shown to be associated with HPV infection [72]. Muenscher et al reported that the patients with no history of tobacco and/or alcohol consumption may also suffer from the laryngeal malignancy. There may be several factors such as individual predisposition, radiation, gastroesophageal reflux, viral infection, dietary factors and environmental influences [73]. The presence of HPV infection in 35.5% of their cases suggests a possible role in the etiology of laryngeal squamous cell carcinoma (LSCC) and supports the role of high-risk types of HPV (16, 18 and 33) in its etiology. HPV infection is not likely to influence survival rates as an independent prognostic factor in patients with laryngeal cancer [74]. Von et al suggested that the broad-spectrum PCR is a reliable method for detection of HPV-DNA [75].

Torrente et al reported that HPV DNA was found in 32% of laryngeal carcinoma biopsies. The genotypes identified were high-risk types, the most frequent being HPV 16. Viral DNA was integrated into the host genome (genotype HPV 16), providing supporting evidence for a role of HPV in the carcinogenic pathway of laryngeal squamous cell carcinoma [76]. Montaldo et al reported that only two HPV genotypes (HPV 16 and HPV 31) were detected in oral carcinoma. [77]. Agrawal et al recently reported that HPV16-positive HNSCC cases were more likely than HPV16-negative cases to have an oral HPV infection detected before and after therapy, consistent with a behavioral and/or biological disposition to infection [78]. Various studies report the differences in the samples and molecular assays utilized in malignant lesions. (Table 3) [87-107].

**Table 3:** Prevalence of HPV in malignant lesions of the Head and neck

**Study**	**Year**	**Types of Lesion**	**Method**	**Positive/cases**	**%**	**HPV type**
Zeuss et al [87]	1991	SCC	ISH	0/15	0	-

Holladay & Gerald [88]	1993	SCC	PCR	7/37	19	16 & 18

Ostwald et al [94]	1994	SCC	PCR/SB	16/26	62	16 (45%), 18 (35%) 6, 11 (15%)

Balaram et al [95]	1995	SCC	PCR	67/91	74	6(13%), 11(20%), 16 (42%)

Cruz et al [96]	1996	SCC	PCR	19/35	55	16(79%)

Wilczynski et al [97]	1998	SCC	PCR	14/21	64	16(80%), 33(10%), 59(10%)

Bustos et al [98]	1999	SCC	ISH	09/33	27	16

van Houten et al [99]	2001	HNSCC	PCR & T-PCR	84	-	High Risk HPV

Kojima et al [100]	2002	SCC	PCR	35/53	66	38

Sugiyama et al [101]	2003	SCC	PCR	30/86	35	16

Smith et al [31]	2004	SCC	PCR	38/201	19	16

Koppikar et al [69]	2005	SCC	PCR	6/102	6	16,18

Slebos et al [102]	2006	SCC	RT-PCR	8/36	22	16

Luo et al [67]	2007	SCC	PCR	11/51	73	-

Zhang et al [103]	2008	SCC	ISH	10/30	33	-

Chuang et al [104]	2008	SCC	RT-PCR	20/59	33.9	16

Simonato et al[105]	2008	SCC	-	5/29	17.2	nested PCR

Richards et al [106]	2009	HNSCC	LOH	-	-	-

Gudleviciene et al [107]	2009	HNSCC	-	-	-	16

Abbreviations: *PCR = Polymerase chain reaction, LOH = Loss of heterozygosity, RT-PCR = Real Time PCR, IHC = Immunohistochemistry, SB = Southern Blotting

### HPV Vaccine

Currently, two prophylactic HPV vaccines, one is quadrivalent 'Gardasil^®^' developed by Merck which protects against HPV types 6, 11, 16 and 18 and another is bivalent Cervarix^® ^vaccine by Glaxo Smith Kline (GSK), protects against HPV types 16 and 18. Prophylactic vaccinations against high-risk HPV types eventually prevent a significant number of carcinomas. Both vaccines are Virus like particle (VLP) vaccines has been developed for primary HPV vaccination and targets HPV 16 and 18, while Gardasil, includes a standard alum adjuvant, also targets HPV 6 and HPV 11. These vaccines are recommended for vaccinating young adolescent girls at or before onset of puberty. Gardasil^® ^(Merck & Co, Bluebell, PA, USA) has gained regulatory approval from U.S.-Food and Drug Administration while Cervarix^® ^(GlaxoSmithKline, Rixensart, Belgium) has been approved in Australia and pending approval from regulatory agencies in the USA. (Table 4)

**Table 4:** Quadrivalent and bivalent human papilloma virus vaccines

**Types of Vaccine**	**Types of HPV**	**Manufacturer**
L1 VLP (Gardasil™) base on recombinant yeast technology	6,11,16 and 18	Merck o., Inc. Whitehouse Station, NJ

L1 VLP (Cevarix™) base on recombinant buculovirus technology	16 and 18	Glaxo Smith Kline (GSK) Rixensart, Belgium

Giorgi Rossi et al reported that the identification of the HPV as the necessary cause of cervical cancer introduced two new tools for prevention: HPV DNA test and vaccine and suggested that vaccine and screening interventions must be integrated in a unique public health program and screening should use less aggressive protocols, to be less expensive and more sustainable and efficient [79]. Marra et al suggested that a female-only vaccination programme is more cost effective compared with the current cytology-based pap smear screening programme, while the cost effectiveness of a male and female vaccination programme is generally less compared with female-only vaccination [80]. The Swedish National Board of Health and Welfare (NBHW) decided that a vaccine that protects against cervical cancer caused by HPV should be included in the childhood vaccination directive as nationwide-programme targeting 12-yearold girls from 2010 as a part of the school-health programme. Currently, vaccination of girls 13–18 years of age is covered by the public insurance [81]. Adams et al suggested that prophylactic HPV vaccination against HPV 16 and 18 has been shown to be highly effective in preventing HPV related malignancy in clinical trials. Newly introduced HPV vaccination program in the UK and elsewhere are ultimately likely to result in a further significant reduction in the incidence and mortality of cervical cancer. These vaccination programs will be most effective in early adolescence when prevalence of HPV infection is low. Consequently, vaccination program in the UK have been initially targeted at 12 to 13-year olds.

In 2007, New South Wales, Australia implemented the National human papillomavirus vaccination program, which provides quadrivalent HPV vaccine free to all women aged 12–26 years. In Australia, favorable estimates of cost effectiveness have supported funding of a 'catch-up' programme to females up to 26 years of age. [82]. Brotherton et al reported that the estimated rate of anaphylaxis for quadrivalent HPV vaccine was significantly higher (IR = 2.6/100 000 doses; 95% CI 1.0–5.3) than identified in comparable school-based delivery of other vaccines like conjugated meningococcal C vaccine (IR = 0.1/100 000 doses; 95% CI 0.003–0.7) However, overall rates were very low and managed appropriately with no serious sequelae [83]. Lawrence et al reported that two deaths were temporally associated with immunization; however, there was no evidence to suggest a causal association. They concluded that despite the low rate of adverse events following immunization in Australia, the passive surveillance system was sufficiently strong to detect safety signals associated with vaccination. Further studies are required before a clean chit can be given to its safety [84].

The Rural Southern areas of North Carolina, USA have accepted the HPV vaccine [85]. Goncalves et al reported that the usefulness of the current HPV vaccine may be considered not only for the cervix but also for prevention of HPV 18 anal infection among immunosuppressed individuals [86]. However in case of oral and oropharyngeal carcinoma, a careful search did not reveal any literature in relation to HPV vaccine.

## Conclusion

HPV may play a role in the carcinogenesis of head and neck malignancies. Molecular mechanisms have been able to recognize its ability to disrupt key cellular elements responsible for the regulation of cell division and apoptosis. Review of literature reveals that HPV may be a risk factor for some head and neck malignancies, but not in all cases. The prevalence of HPV in virus-related benign lesions as well as malignancies has been confirmed by many molecular techniques, among those, PCR is the most sensitive method. For the confirmation of the role of HPV in head and neck malignancies, large population studies are necessary in an assortment of clinical settings.

## Competing interests

The authors declare that they have no competing interests.

## Authors' contributions

All authors read and approved the final manuscript.
